# Identification of *Trueperella bernardiae* isolated from peking ducks (*Anas platyrhynchos domesticus*) by phenotypical and genotypical investigations and by a newly developed loop-mediated isothermal amplification (LAMP) assay

**DOI:** 10.1007/s12223-021-00927-4

**Published:** 2021-11-15

**Authors:** Marwa F. E. Ahmed, Mazen Alssahen, Christoph Lämmler, Bernd Köhler, Martin Metzner, Madeleine Plötz, Amir Abdulmawjood

**Affiliations:** 1 Hygiene and Zoonoses Department, Faculty of Veterinary Medicine, Mansoura University, Elgomhoria Street 60, Mansoura, 35516 Egypt; 2 Institute for Animal Nutrition, University of Veterinary Medicine Hannover, Foundation, Bischofsholer Damm 15, 30173 Hannover, Germany; 3 Institut für Hygiene und Infektionskrankheiten der Tiere, Justus-Liebig-Universität Gießen, Frankfurterstraße 85-91, 35392 Gießen, Germany; 4 Ripac-Labor GmbH, Am Mühlenberg 11, 14476 Potsdam, Germany; 5 Institute of Food Quality and Food Safety, University of Veterinary Medicine Hannover, Bischofsholer Damm 15, 30173 Hannover, Germany

## Abstract

*Trueperella* (*T.*) *bernardiae* is a well-known bacterial pathogen in infections of humans, rarely in animals. In the present study, five *T*. *bernardiae* isolates, isolated from five Peking ducks of four different farms, were identified by phenotypic properties, by matrix-assisted laser desorption/ionization time-of-flight mass spectrometry (MALDI-TOF MS) analysis, and genotypically by sequencing the 16S ribosomal RNA (rRNA) gene, the superoxide dismutase A encoding gene *sodA*, and the glyceraldehyde-3-phosphate dehydrogenase encoding gene *gap*. In addition, the *T*. *bernardiae* isolates could be identified with a newly developed loop-mediated isothermal amplification (LAMP) assay based on the gyrase encoding housekeeping gene *gyrA*. All these tests clearly identified the *T*. *bernardiae* isolates to the species level. However, the detection of the specific gene *gyrA* with the newly designed LAMP assay appeared with a high sensitivity and specificity, and could help to identify this bacterial species in human and animal infections in future. The importance of the *T*. *bernardiae* isolates for the clinical condition of the ducks and for the problems at farm level remains unclear.

## Introduction

*Trueperella* (*T.*) *bernardiae* is a gram-positive, non-motile, and facultatively anaerobic coccobacillus which was originally identified as a coryneform group 2 bacterium (Na’Was et al. 1987). Eight years later, this bacterium was recognized as *Actinomyces bernardiae* (Funke et al. 1995) and then reclassified as part of the genus *Arcanobacterium* as *Arcanobacterium bernardiae* (Ramos et al. 1997). According to a proposal of Yassin et al. (2011), *Arcanobacterium bernardiae* was finally classified to the newly described genus *Trueperella* as *T. bernardiae*, together with *T. pyogenes*, *T. bialowiezensis*, *T. bonasi*, and *T. abortisuis*.

In humans, *T. bernardiae* was first described as an opportunistic pathogen and later could be identified alone or in combination with other bacteria as causing joint infections (Gilarranz et al. 2016; Gowe et al. 2018), urinary tract infections (Lepargneur et al. 1998), abscesses (Parha et al. 2015; VanGorder et al. 2016; Calatrava et al. 2019; Pan et al. 2019), wound infections (Weitzel et al. 2011; Rattes et al. 2016; Cobo et al. 2017), a diabetic foot infection (Schneider et al. 2015), bacteremia (Otto et al. 2013; Roh et al. 2019), and septic thrombophlebitis (Lawrence et al. 2018).

The first characterization of *T. bernardiae* of animal origin (3-day-old piglet) was made by Hijazin et al. (2012c). In a second case, Arnafia et al. (2017) described a *T. bernardiae* strain recovered from a purulent thelitis of a 12-year-old male dog.

These previously isolated *T. bernardiae* isolates of animal origin were identified and further characterized by matrix-assisted desorption ionization–time of flight mass spectrometry (MALDI-TOF MS) and by sequencing various genomic targets (Hijazin et al. 2012c; Arnafia et al. 2017).

At present, no further data are available concerning the isolation of *T. bernardiae* from animals or animal infections.

In the present investigation, five *T. bernardiae* isolates recovered from post mortem samples of Peking ducks (*Anas platyrhynchos domesticus*) could be identified phenotypically and genotypically, and with a newly described loop-mediated isothermal amplification (LAMP) assay based on the housekeeping gene *gyrA*.

## Material and methods

### Bacterial strains

The five *T. bernardiae* isolates investigated in the present study (*T. bernardiae* D12-0613-1-4-3, *T. bernardiae* D13-1622-5-3-2, *T. bernardiae* D14-1577-4-8-1, *T*. *bernardiae* D13-1772-748-1-2, *T. bernardiae* D14-1481-2029-1-1) were isolated from five Peking ducks (*Anas platyrhynchos domesticus*) from three different farms in Germany (*n* = 3) and one in Thailand (*n* = 2). The Geman samples were collected from the heart, lung, and joint, while the Thailand samples were delivered as swabs after post-mortem examination; unfortunately, the source organs were not defined. All five isolates were collected during post-mortem examination in the period between 2012 and 2014. Further details about the five isolates are given in Table 1. The bacterial cultivation and a preliminary identification of the bacteria were performed at Ripac-Labor GmbH, Potsdam, Germany. The bacterial culturing of the *T. bernardiae* isolates was carried out on sheep blood agar plates (Oxoid GmbH, Wesel, Germany) for 48 h at 37 °C under a microaerophilic gas atmosphere using a candle jar.

**Table 1:** Data on the five *T. bernardiae* isolates recovered from Peking ducks investigated in the present study

Strain code	Farm/country	Sample drawing	Sample source	Further information
*T. bernardiae* D12-0613-1-4-3	A/G	07/05/2012	Post-mortem/heart; no pathological findings	Accompanying bacteria: *Corynebacterium* spp., *Aerococcus viridans, Escherichia* (*E.) coli*
*T. bernardiae* D13-1622-5-3-2	B/G	02/10/2013	Post-mortem/lung edema	Accompanying bacteria: *Aspergillus fumigatus, Corynebacterium confusum, Coenonia anatina*; increased mortality and joint infections at farm level
*T. bernardiae* D13-1772-748-1-2	C/T	25/10/2013	Post-mortem	Accompanying bacteria: *Globicatella sulfidifaciens*
*T. bernardiae* D14-1481- 2029-1-1	C/T	20/08/2014	Post-mortem	n.d
*T. bernardiae* D14-1577-4-8-1	D/G	05/09/2014	Post-mortem/joint infection	Accompanying bacteria: *Trueperella pyogenes, E. coli*; Increased mortality at farm level

*G* Germany, *T* Thailand, *n.d.* no data available

### Phenotypic identification

A phenotypic identification was performed using conventional cultural and biochemical assays as previously shown (Hassan et al. 2009; Ülbegi-Mohyla et al. 2009) and with the API-Coryne test system (BioMérieux Deutschland GmbH, Nürtingen, Germany) in accordance with the manufacturer’s instructions. Furthermore, the bacterial isolates were identified by MALDI-TOF MS using a Microflex LT (Bruker Daltonik GmbH, Bremen, Germany) instrument following the manufacturer’s instructions using the direct transfer method. Briefly, one microbial colony was first smeared in duplicate onto spots of the MALDI MSP 96 target plate (MicroScout Target plate; Bruker Daltonik GmbH) with sterile toothpicks. The air-dried bacteria were overlaid with 1µL of an α-cyan 4-hydroxycinnamic acid matrix solution (HCCA, in 50% acetonitrile and 2.5% trifluoroacetic acid in pure water), followed by drying and loading into the mass spectrometer. The analysis of the spectra was carried out by MBT Compass Explorer 4.1 software (Bruker Daltonik GmbH).

### DNA extraction

The genomic DNA of the five isolates, type strain *T. bernardiae* DSM 9152^ T^, and various other strains of genus *Trueperella* and genus *Arcanobacterium* (*A.*) were extracted using the DNeasy Blood and Tissue kit (Qiagen GmbH, Hilden, Germany), in conformance with the manufacturer’s instructions. The concentration and purity of DNA were measured by means of a NanoDrop spectrophotometer (ND1000; Thermo Fisher Scientific GmbH, Dreieich, Germany).

### Sequencing the molecular targets

The five *T. bernardiae* isolates were also investigated by sequencing the following molecular targets: 16S rRNA gene, superoxide dismutase A encoding gene *sodA*, and glyceraldehyde-3-phosphate dehydrogenase encoding gene *gap*. The sequences of the oligonucleotide primers and the PCR conditions were used as previously described for 16S rRNA gene (Hassan et al. 2009), gene *sodA* (Hijazin et al. 2011, 2012c), and gene *gap* (Wickhorst et al. 2019). The PCR products were purified and sequenced by Eurofins Genomics Germany GmbH (Ebersberg, Germany). The obtained sequences of the different genes of the *T. bernardiae* isolates were aligned and further analyzed using the clustal w method of the MegAlign program version 15 (DNASTAR, Inc., Madison, WI, USA) and compared with the nucleotide sequences of the targets 16S rRNA gene, *sodA*, and *gap* of type strain *T. bernardiae* DSM 9152^ T^, and type strain of *T. pyogenes* DSM 20630^ T^ obtained from the NCBI GenBank, and for control purposes from *A. haemolyticum* DSM 20595^ T^ also obtained from the NCBI GenBank.

### LAMP assay

#### Design of oligonucleotide primers for LAMP assay

Oligonucleotide primers for the *T. bernardiae-*specific LAMP assay were developed using the gyrase subunit A encoding gene *gyrA* of *T. bernardiae* (LNIZ01000002).

The LAMP primers (forward outer primer *gyrA*-F3, backward outer primer *gyrA*-B3, forward inner primer *gyrA*-FIP, backward inner primer *gyrA*-BIP, forward loop primer *gyrA*-LoopF, and backward loop primer *gyrA*-LoopB) were designed using the LAMP designer software (PREMIER Biosoft, San Francisco, CA, USA) (Table 2). The oligonucleotide primers were synthesized by Eurofins Genomics.

**Table 2:** Oligonucleotide primer sequences of gyrase subunit A encoding gene *gyrA* used for development of the *T. bernardiae* LAMP assay

Designation	Sequences 5′- 3′	Primer length (bp)	Melting temperature (°C)
*gyrA*-F3	CACCAGGTAGAGGTCATCA	19	56.7
*gyrA* -B3	TCCTCGACGATCTTCTGC	18	56.0
*gyrA* -FIP	GCCGGATGAGGGCAATGAGAAGAGCGCCTCATGATC	36	˃75
*gyrA* -BIP	CGGGCTCATCGAACTGCTCTGCATGGCGAGGATATG	36	˃75
*gyrA* -LoopF	CTCGTCCAGCATGTCGAG	18	58.2
*gyrA* -LoopB	CGCGATCAACGAGATCCA	18	56.0

#### LAMP reaction and amplification conditions

In accordance with the manufacturer’s instructions, the LAMP assay based on gene *gyrA* was carried out with the five *T. bernardiae* isolates, type strain *T. bernardiae* DSM 9152^ T^, and with control strains of genus *Trueperella* and closely related genus *Arcanobacterium*. A total volume of 25 µL for each reaction included 15 µL GspSSD isothermal master mix (ISO-001) (OptiGene Ltd., Horsham, UK) and 2.5 µL primer mix (ISO-001; OptiGene Ltd.), *gyrA-* F3 primer, and *gyrA*-B3 primer with a final concentration equivalent to 0.2 µmol/L, *gyrA*-FIP primer, and *gyrA*-BIP primer with final concentration equivalent to 0.8 µmol/L and *gyrA*-LoopF Primer and *gyrA*-LoopB Primer with a final concentration equivalent to 0.4 µmol/L. Subsequently, 5 µL DNA was added as a template. The LAMP assay was run at 70 °C for 20 min with a melting curve analysis step (annealing curve 98 to 80 °C ramping at 0.05 °C/s) in a real-time fluorometer GenieII® (OptiGene Ltd.).

#### Analytical sensitivity and specificity of the LAMP assay

Determination of the analytic sensitivity of the LAMP assay was performed seven times using a serially diluted DNA (10^−1^–10^−6^) isolated from type strain *T. bernardiae* DSM 9152^ T^ in AE buffer (10 mM Tris–Cl, 0.5 mM EDTA; pH 9.0) with the conditions mentioned above. DNA isolation and concentration were performed as stated above. The amount of DNA ranged from 3.0 ng/µL (10^−0^) to 3.0 fg/µL (10^−6^) bacterial DNA. The colony-forming unit (cfu/mL) was subsequently estimated.

The specificity of the LAMP assay was determined using the DNA of *T. bernardiae* DSM 9152^ T^ and closely related species of genus *Trueperella* and *Arcanobacterium*. These included *T. pyogenes* DSM 20630^ T^, *T. pyogenes* DSM 20594, *T. pyogenes* 59/11, *T. abortisuis* DSM 19515^ T^, *T. bialowiezensis* DSM 17162^ T^, *T. bonasi* DSM 17163^ T^, *A. hippocoleae* DSM 15539^ T^, *A. pluranimalium* DSM 13483^ T^, and *A. phocae* DSM 10002^ T^. The LAMP assay was performed with the optimized LAMP protocol with a run-time of 20 min.

## Results and discussion

The phenotypic properties of the five *T. bernardiae* isolates of duck origin investigated in the present study were almost identical to those of type strain *T. bernardiae* DSM 9152^ T^, and to previously characterized *T. bernardiae* strains of pig and dog origin (Hijazin et al. 2012c; Arnafia et al. 2017). All *T. bernardiae* isolates gave positive reactions for pyrazinamidase, pyrrolidonyl arylamidase, and α-glucosidase, and reacted negatively in nitrate reduction and for alkaline phosphatase, β-glucuronidase, β-galactosidase, and N-acetyl-β-glucosaminidase. Also, all isolates did not hydrolyze esculin, urea, and gelatine. The isolates also fermented D-glucose, except *T. bernardiae* D14-1481-2029-1-1 and type strain *T. bernardiae* DSM 9152^ T^, D-ribose, D-maltose, and glycogen, but not D-xylose, D-mannitol, D-lactose, and D-saccharose. In addition, all isolates showed a negative catalase reaction (Table 3).

**Table 3:** Biochemical properties of the five *T. bernardiae* isolates investigated in the present study and *T. bernardiae* DSM 9152^ T^

**Biochemical properties**	***T. bernardiae* D12-0613–1-4–3**	***T. bernardiae* D13-1622–5-3–2**	***T. bernardiae* D13-1772–748-1–2**	***T. bernardiae* D14-1481- 2029–1-1**	***T. bernardiae* D14-1577–4-8–1**	***T. bernardiae* DSM 9152**^** T**^
Nitrate reduction	−	−	−	−	−	−
Pyrazinamidase	+	+	+	+	+	+
Pyrrolidonyl Arylamidase	+	+	+	+	+	+
Alkaline phosphatase	−	−	−	−	−	−
α-Glucuronidase	−	−	−	−	−	−
β-Galactosidase	−	−	−	−	−	−
β-Glucosidase	+	+	+	+	+	+
N-Acetyl- β -glucosaminidase	−	−	−	−	−	−
Esculin	−	−	−	−	−	−
Urease	−	−	−	−	−	−
Gelatine	−	−	−	−	−	−
**Fermentation**						
Glucose	+	+	+	−	+	−
Ribose	+	+	+	+	+	+
Xylose	−	−	−	−	−	−
Mannitol	−	−	−	−	−	−
Maltose	+	+	+	+	+	+
Lactose	−	−	−	−	−	−
Saccharose	−	−	−	−	−	−
Glycogen	+	+	+	+	+	+
Catalase	−	−	−	−	−	−
*T. bernardiae* identification (%) according to Api- Coryne test system	99.7	99.7	99.7	99.9	99.7	99.9

+ positive reaction, − negative reaction, ^**T**^type strain

With the additionally performed MALDI-TOF MS analysis, all five isolates were identified to the species level as *T. bernardiae* with log-score values varying between 1.87 and 2.2 (data not shown). MALDI-TOF MS appeared to be a fast, accurate, and less expensive tool for microbial identification of bacteria, viruses, and fungi (Singhal et al. 2015), also including *T. bernardiae* (Hijazin et al. 2012a) and various other species of genera *Trueperella* and *Arcanobacterium* (Hijazin et al. 2012a, b).

**Fig. 1:**
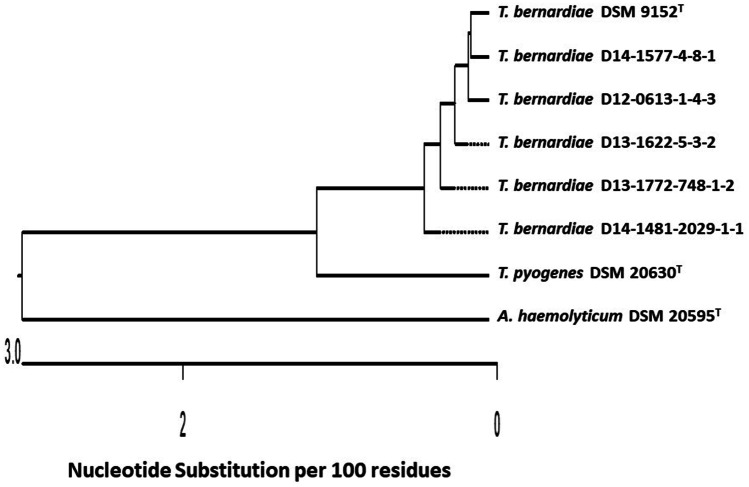
Phylogenetic analysis based on nucleotide sequences of 16S rRNA gene of the five investigated *T. bernardiae* isolates isolated from Peking ducks, type strain *T. bernardiae* DSM 9152^ T^, and closely related *T. pyogenes* DSM 20630^ T^ and *A. haemolyticum* DSM 20595^ T^ obtained from NCBI GenBank

The five *T. bernardiae* isolates in the current study were additionally identified genotypically by amplification and sequencing of the 16S rRNA gene. The nucleotide sequence of *T. bernardiae* D12-0613-1-4-3 (GenBank accession number: MT364890), *T. bernardiae* D13-1622-5-3-2 (MT364891), *T. bernardiae* D13-1772-748-1-2 (MT364892), *T. bernardiae* D14-1481-2029-1-1 (MT364893), and *T. bernardiae* D14-1577-4-8-1 (MT364894) were compared with type strain *T. bernardiae* DSM 9152^ T^ (HE653979), *T. pyogenes* DSM 20630^ T^ (X79225), and *Arcanobacterium* (*A.*) *haemolyticum* DSM 20595^ T^ (AJ234059). The nucleotide sequence data of *T. bernardiae* D12-0613-1-4-3, *T. bernardiae* D13-1622-5-3-2, *T. bernardiae* D13-1772-748-1-2, *T. bernardiae* D14-1481-2029-1-1, and *T. bernardiae* D14-1577–4-8–1 revealed a sequence homology of 99.7, 99.7, 99.2, 99.0, and 99.7% with type strain *T. bernardiae* DSM 9152^ T^, respectively (Fig. 1). The 16S rRNA gene sequence similarities of the five *T. bernardiae* to *T. pyogenes* DSM 20630^ T^ and *A. haemolyticum* DSM 20595^ T^ were equal or less than 98.1 and 94.8%, respectively (Fig. 1).

**Fig. 2:**
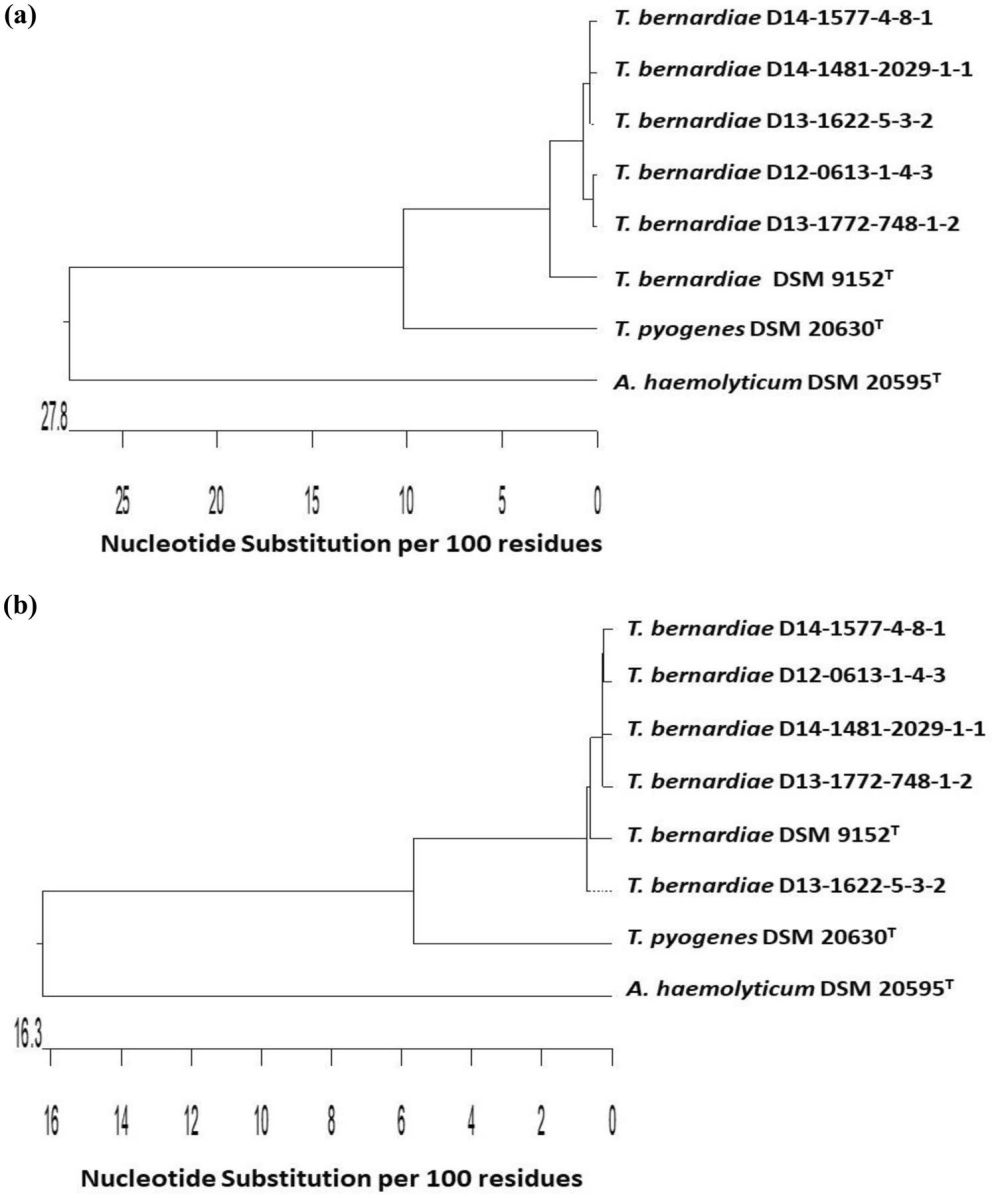
Dendrogram analysis of superoxide dismutase A encoding gene *sodA* (**a**) and glyceraldehyde-3-phosphate dehydrogenase encoding gene *gap* (**b**) of the five *T. bernardiae* isolates isolated from Peking ducks, type strain *T. bernardiae* DSM 9152^ T^, and closely related *T. pyogenes* DSM 20630^ T^ and *A. haemolyticum* DSM 20595^ T^ obtained from NCBI GenBank

The five *T. bernardiae* isolates could be further characterized by PCR-mediated amplification of the genes *sodA* and *gap*. The sequences of gene *sodA* of *T. bernardiae* D12-0613–1-4–3 (MT410971), *T. bernardiae* D13-1622–5-3–2 (MT410972), *T. bernardiae* D13-1772–748-1–2 (MT410973), *T. bernardiae* D14-1481–2029-1–1 (MT410974), and *T. bernardiae* D14-1577–4-8–1 (MT410975) resulted in sequence similarities of 95.8, 94.7, 95.5, 93.8, and 95.0% with the *sodA* gene of type strain *T. bernardiae* DSM 9152^ T^ (AM989465), respectively, while the similarity within the five isolates was between 99.0 and 100% (Fig. 2a).

The additionally investigated *gap* genes of *T. bernardiae* D12-0613–1-4–3 (MT410966), *T. bernardiae* D13-1622–5-3–2 (MT410967), *T. bernardiae* D13-1772–748-1–2 (MT410968), *T. bernardiae* D14-1481–2029-1–1 (MT410969), and *T. bernardiae* D14-1577–4-8–1 (MT410970) showed sequence similarities of 98.5, 98.8, 98.8, 99.0, and 99.0% with the *gap* gene of type strain *T. bernardiae* DSM 9152^ T^ (HF947287), respectively, while the similarity within the five isolates was between 99.0 and 99.8% (Fig. 2b). The gene sequences of the *sodA* and *gap* genes of the five *T. bernardiae* isolates showed a clear difference to the control strains *T. pyogenes* DSM 20630^ T^ (AM949566 and HF947285, respectively) and *A. haemolyticum* DSM 20595^ T^ (AM983534 and CP002045, respectively) (Fig. 2a, b). All three mentioned genomic targets were already used to characterize various species of genus *Trueperella*, also including *T. bernardiae* (Hassan et al. 2009; Hijazin et al. 2011, 2012c; Arnafia et al. 2017).

The additionally used *T. bernardiae gyrA*-specific LAMP assay could successfully be used to identify the species-specific gene *gyrA* of all five *T. bernardiae* isolates in the present investigation. This newly established assay demonstrated a specificity for *T. bernardiae* DSM 9152^ T^ with an annealing temperature between 91.9 and 92.4 °C. No cross-reactivity with any other related species of genus *Trueperella* or genus *Arcanobacterium* could be observed (Table 4).

**Table 4:** Specificity of the *T.* *bernardiae* LAMP assay based on gene *gyrA* for *T. bernardiae* DSM 9152^ T^, the five *T.* *bernardiae* isolates of duck origin, and other closely related species of genus *Trueperella* and *Arcanobacterium*

Species and strain number	Detection time mm:ss	Melting temperature (°C)
*T. bernardiae* DSM 9152^ T^	11:45	91.9
*T. bernardiae* D12-0613–1-4–3	14:15	92.0
*T. bernardiae* D13-1622–5-3–2	13:15	92.3
*T. bernardiae* D13-1772–748-1–2	13:30	92.3
*T. bernardiae* D14-1481- 2029–1-1	13:00	92.4
*T. bernardiae* D14-1577–4-8–1	13:15	92.4
*T. pyogenes* DSM 20630^⊤^	–	–
*T. pyogenes* DSM 20594	–	–
*T. pyogenes* 59/11	–	–
*T. abortisuis* DSM 19515 T	–	–
*T. bialowiezensis* DSM 17162 T	–	–
*T. bonasi* DSM 17163 T	–	–
*A. hippocoleae* DSM 15539 T	–	–
*A. pluranimalium* DSM 13483^ T^	–	–
*A. phocae* DSM 10002 T	–	–

*DSM* Deutsche Sammlung von Mikroorganismen und Zellkulturen

**Fig. 3:**
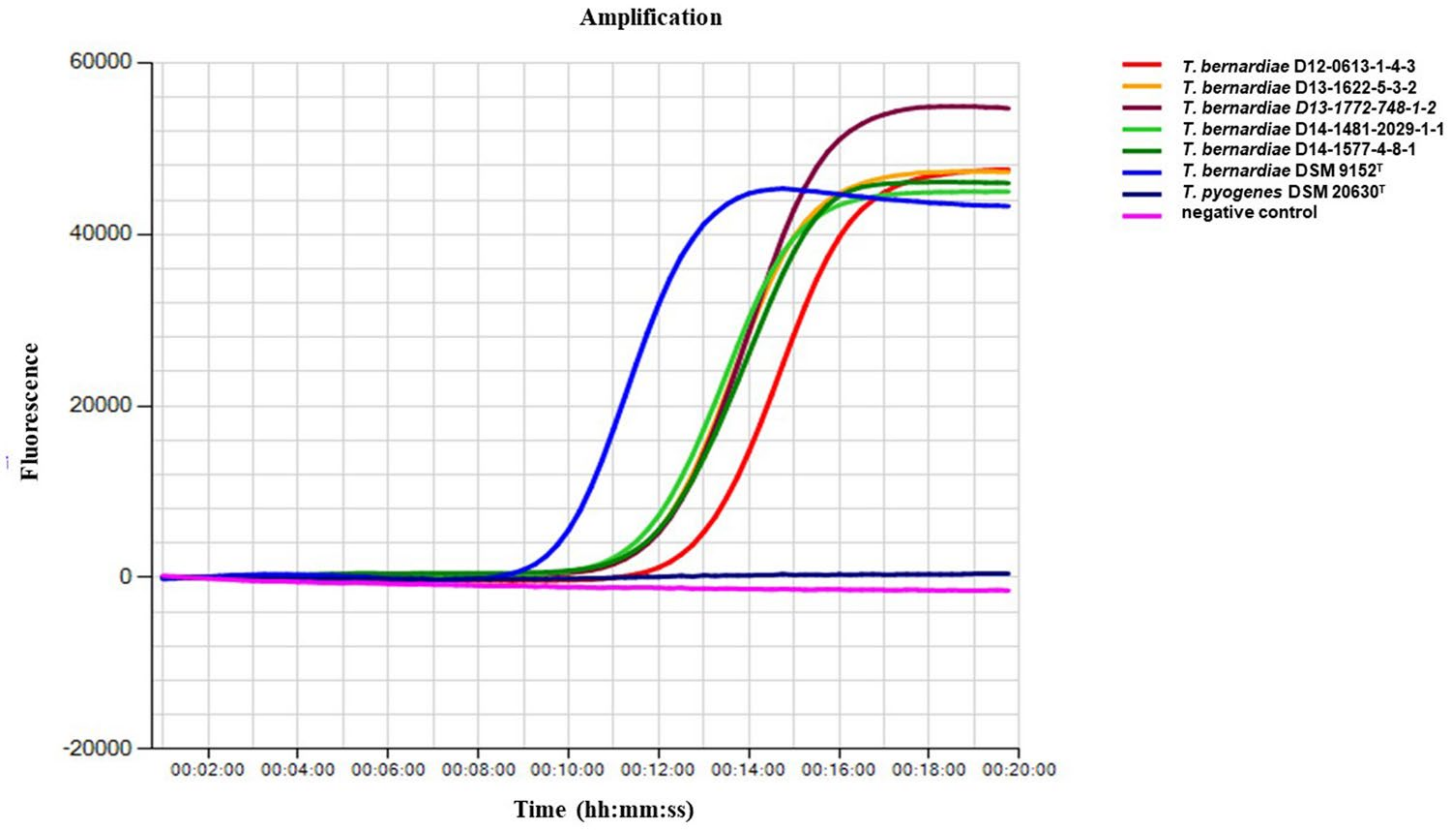
Positive LAMP assay of the five *T. bernardiae* isolates *T. bernardiae* D12-0613–1-4–3, *T. bernardiae* D13-1622–5-3–2, *T. bernardiae* D13-1772–748-1–2, *T. bernardiae* D14-1481–2029-1–1, *T. bernardiae* D14-1577–4-8–1 obtained from Peking ducks, *T. bernardiae* DSM 9152^ T^, and as LAMP negative control *T. pyogenes* DSM 20630^ T^ and nuclease free water as negative control

The developed LAMP assay provided an analytic sensitivity of 30 fg/μL with a mean detection time between 00:08:58 (3.0 ng/μL) and 00:18:35 min (30 fg/μL) (Table 5). For the DNA concentration at 30 fg/μL (10^−5^), the assay gave positive results in six of seven reactions (85.7%), whereas for DNA concentration at 3.0 fg/µL (10^−6^), the positive assay resulted in two of seven replicates (28.6%). The results of the *T. bernardiae gyrA* LAMP assay are shown in Fig. 3 and Table 4.

**Table 5:** Detection time and annealing temperature of the LAMP assay using bacterial serial dilutions of type strain *T. bernardiae* DSM 9152^ T^

*T. bernardiae* DSM 9152^ T^		Serial dilution					
	cfu/mL	10^−1^	10^−2^	10^−3^	10^−4^	10^−5^	10^−6^
Detection time mean (mm:ss)	2.84 × 10^8^	08:58	09:15	11:39	15:56	18:35	17:15
SD ( ±) detection time		00:49	02:52	01:29	04:25	08:32	06:00
Annealing temp. (°C) mean		91.9	91.8	91.9	91.8	91.8	91.8
SD ( ±) annealing		0.15	0.12	0.22	0.19	0.22	0.22

The application of LAMP assays as being a rapid and reliable method for detecting species of genus *Trueperella* and *Arcanobacterium* has been previously published. In 2013, Zhang et al. developed a LAMP assay using the gene encoding pyolysin, the *plo* gene, for a specific identification of *T. pyogenes* (Zhang et al. 2013). Furthermore, a *pla* LAMP assay was used for identifying *A. pluranimalium* (Abdulmawjood et al. 2015), and a *cpn60* LAMP assay for identifying *T. pyogenes* from different animal origins (Abdulmawjood et al. 2016; Ahmed et al. 2020; Alssahen et al. 2020).

The present study gives a reliable phenotypic and genotypic characterization of *T. bernardiae* of duck origin, also including a newly developed LAMP assay. To our knowledge, the study gives the first detailed characterization of this bacterial species isolated from Peking ducks. However, the pathogenic importance of *T. bernardiae*, which was partly isolated together with various other bacteria from apparently healthy animals, for the high mortality rate or joint infections of the Peking ducks at farm level remains unclear. The described LAMP assay might help to identify this bacterial species in future and might elucidate the role this species plays in human and animal infections.

## Data Availability

The data that support the findings of this study are available on NCBI’s Genbank and are accessible through the accession numbers listed in the manuscript.

## References

[CR1] Abdulmawjood A, Wickhorst J, Hashim O, Sammra O, Hassan AA, Alssahen M, Lämmler C, Prenger-Berninghoff E, Klein G (2016). Application of a loop-mediated isothermal amplification (LAMP) assay for molecular identification of *Trueperella pyogenes* isolated from various origins. Mol Cell Probe.

[CR2] Abdulmawjood A, Wickhorst J, Sammra O, Lämmler C, Foster G, Wragg PN, Prenger-Berninghoff E, Klein G (2015). Development of a loop-mediated isothermal amplification (LAMP) assay for rapid and sensitive identification of* Arcanobacterium pluranimalium*. Mol Cell Probe.

[CR3] Ahmed MFE, Alssahen M, Lämmler C, Eisenberg T, Plötz M, Abdulmawjood A (2020). Studies on *Trueperella pyogenes* isolated from an okapi (*Okapia johnstoni*) and a royal python (*Python regius*). BMC Vet Res.

[CR4] Alssahen M, Peters M, Rau J, Hassan AA, Sammra O, Lämmler C, Prenger-Berninghoff E, Plötz M, Abdulmawjood A (2020) Phenotypic and genotypic approach to characterize a *Trueperella pyogenes* strain isolated from an Eurasian Lynx (*Lynx lynx*). Berl Münch Tierärzt Wochenschr 133. 10.2376/0005-9366-19037

[CR5] Arnafia W, Ningrum SG, Sammra O, Alssahen M, Wickhorst J-P, Lämmler C, Prenger-Berninghoff E, Timke M, Abdulmawjood A (2017). Phynotypic and genotypic analysis of a *Trueperella bernardiae* strain isolated from a dog. Veterinaria (sarajevo).

[CR6] Calatrava E, Borrego J, Cobo F (2019). Breast abscess due to *Trueperella bernardiae* and *Actinotignum sanguinis*. Rev Esp Quim.

[CR7] Cobo F, Rodriguez-Granger J, Sampedro A, Gutierrez-Fernandez J, Navarro-Mari JM (2017). Two rare cases of wound infections caused by *Trueperella bernardiae*. Jpn J Infect Dis.

[CR8] Funke G, Ramos CP, Fernandez-Garayzabal JF, Weiss N, Collins MD (1995). Description of human-derived centers for disease control coryneform group 2 bacteria as *Actinomyces bernardiae* sp. nov. Int J Syst Evol Microbiol.

[CR9] Gilarranz R, Chamizo F, Horcajada I, Bordes-Benitez A (2016). Prosthetic joint infection caused by *Trueperella bernardiae*. J Infect Chemother.

[CR10] Gowe I, Parsons C, Best M, Parsons E, Prechter S, Vickery S (2018). Successful treatment of olecranon bursitis caused by *Trueperella bernardiae*: importance of environmental exposure and pathogen identification. Case Rep Infect Dis.

[CR11] Hassan AA, Ülbegi-Mohyla H, Kanbar T, Alber J, Lämmler C, Abdulmawjood A, Zschöck M, Weiss R (2009). Phenotypic and genotypic characterization of *Arcanobacterium haemolyticum* isolates from infections of horses. J Clin Microbiol.

[CR12] Hijazin M, Alber J, Lämmler C, Weitzel T, Hassan A, Timke M, Kostrzewa M, Prenger-Berninghoff E, Zschöck M (2012). Identification of *Trueperella* (*Arcanobacterium*) *bernardiae* by matrix-assisted laser desorption/ionization time-of-flight mass spectrometry analysis and by species-specific PCR. J Med Microbiol.

[CR13] Hijazin M, Hassan AA, Alber J, Lämmler C, Timke M, Kostrzewa M, Prenger-Berninghoff E, Zschöck M (2012). Evaluation of matrix-assisted laser desorption ionization-time of flight mass spectrometry (MALDI-TOF MS) for species identification of bacteria of genera *Arcanobacterium* and *Trueperella*. Vet Microbiol.

[CR14] Hijazin M, Metzner M, Erhard M, Nagib S, Alber J, Lämmler C, Hassan AA, Prenger-Berninghoff E, Zschöck M (2012). First description of *Trueperella* (*Arcanobacterium*) *bernardiae* of animal origin. Vet Microbiol.

[CR15] Hijazin M, Ülbegi-Mohyla H, Alber J, Lämmler C, Hassan AA, Abdulmawjood A, Prenger-Berninghoff E, Weiss R, Zschöck M (2011). Molecular identification and further characterization of *Arcanobacterium pyogenes* isolated from bovine mastitis and from various other origins. J Dairy Sci.

[CR16] Lawrence C, Waseem S, Newsholme W, Klein J (2018). *Trueperella bernardiae*: an unusual cause of septic thrombophlebitis in an injection drug user. New Microbes New Infect.

[CR17] Lepargneur J, Heller R, Soulie R, Riegel P (1998). Urinary tract infection due to *Arcanobacterium bernardiae* in a patient with a urinary tract diversion. Eur J Clin Microbiol Infect Dis.

[CR18] Na’Was T E, Hollis D, Moss C W, Weaver R E, (1987). Comparison of biochemical, morphologic, and chemical characteristics of centers for disease control fermentative coryneform groups 1, 2, and A-4. J Clin Microbiol.

[CR19] Otto MP, Foucher B, Lions C, Dardare E, Gerome P (2013). *Trueperella bernardiae* soft tissue infection and bacteremia. Med Maladies Infect.

[CR20] Pan J, Ho AL, Pendharkar AV, Sussman ES, Casazza M, Cheshier SH, Grant GA (2019). Brain abscess caused by *Trueperella bernardiae* in a child. Surg Neurol Int.

[CR21] Parha E, Alalade A, David K, Kaddour H, Degun P, Namnyak S (2015). Brain abscess due to *Trueperella bernardiae*. Br J Neurosurg.

[CR22] Ramos CP, Foster G, Collins MD (1997). Phylogenetic analysis of the genus *Actinomyces* based on 16S rRNA gene sequences: description of *Arcanobacterium phocae* sp. nov., *Arcanobacterium bernardiae* comb. nov., and *Arcanobacterium pyogenes *comb. nov. Int J Syst Evol Microbiol.

[CR23] Rattes AL, Araujo MR, Federico MP, Magnoni CD, Neto PA, Furtado GH (2016). *Trueperella bernardiae*: first report of wound infection post laparoscopic surgery. Clin Case Rep.

[CR24] Roh J, Kim M, Kim D, Yong D, Lee K (2019). First case of *Trueperella bernardiae* bacteremia in an immunocompromised patient in Korea. Ann Lab Med.

[CR25] Schneider UV, Ekenberg C, Sode N, Knudsen JD (2015) A case of diabetic foot ulcers complicated by severe infection and sepsis with *Trueperella bernardiae*. JMM Case Reports 2(1). 10.1099/jmmcr.0.000006

[CR26] Singhal N, Kumar M, Kanaujia PK, Virdi JS (2015) MALDI-TOF mass spectrometry: an emerging technology for microbial identification and diagnosis. Front Microbiol 6:791. 10.3389/fmicb.2015.00791 10.3389/fmicb.2015.00791 PMC4525378 26300860

[CR27] Ülbegi-Mohyla H, Hassan AA, Kanbar T, Alber J, Lämmler C, Prenger-Berninghoff E, Weiss R, Siebert U, Zschöck M (2009). Synergistic and antagonistic hemolytic activities of bacteria of genus *Arcanobacterium* and CAMP-like hemolysis of *Arcanobacterium phocae* and *Arcanobacterium haemolyticum* with *Psychrobacter phenylpyruvicus*. Res Vet Sci.

[CR28] VanGorder BRF, Ahmed SS, Rawling RA, Granato PA (2016). *Trueperella bernardiae* abscess infection: a case report. Clin Microbiol Newsl.

[CR29] Weitzel T, Braun S, Porte L (2011). *Arcanobacterium bernardiae* bacteremia in a patient with deep soft tissue infection. Surg Infect (larchmt).

[CR30] Wickhorst JP, Hassan AA, Sammra O, Alssahen M, Lämmler C, Prenger-Berninghoff E, Naggert M, Timke M, Rau J, Abdulmawjood A (2019). First report on the isolation of *Trueperella abortisuis* from companion animals. Res Vet Sci.

[CR31] Yassin A, Hupfer H, Siering C, Schumann P (2011) Comparative chemotaxonomic and phylogenetic studies on the genus *Arcanobacteriem Collins* et al. 1982 emend. Lehnen et al. 2006: proposal for *Trueperella* gen. nov. and emended description of the genus *Arcanobacterium*. Int J Syst Evol Microbiol 61:1265–1274 10.1099/ijs.0.020032-0 20622055

[CR32] Zhang WL, Meng XL, Wang JW (2013). Sensitive and rapid detection of *Trueperella pyogenes* using loop-mediated isothermal amplification method. J Microbiol Meth.

